# Design and Synthesis Strategy of MXenes-Based Anode Materials for Sodium-Ion Batteries and Progress of First-Principles Research

**DOI:** 10.3390/molecules28176292

**Published:** 2023-08-28

**Authors:** Dan Su, Hao Zhang, Jiawei Zhang, Yingna Zhao

**Affiliations:** 1 Hebei Province Laboratory of Inorganic Nonmetallic Materials, College of Material Science and Engineering, North China University of Science and Technology, Tangshan 063210, China; 15612578603@163.com (D.S.); zhangpepsi007@163.com (J.Z.); 2 College of Clinical Medicine, North China University of Science and Technology, Tangshan 063210, China; haozhang0823@foxmail.com; 3 Hebei (Tangshan) Ceramic Industry Technology Research Institute, Tangshan 063007, China

**Keywords:** MXene, sodium-ion battery, negative electrode materials, density functional theory

## Abstract

MXenes-based materials are considered to be one of the most promising electrode materials in the field of sodium-ion batteries due to their excellent flexibility, high conductivity and tuneable surface functional groups. However, MXenes often have severe self-agglomeration, low capacity and unsatisfactory durability, which affects their practical value. The design and synthesis of advanced heterostructures with advanced chemical structures and excellent electrochemical performance for sodium-ion batteries have been widely studied and developed in the field of energy storage devices. In this review, the design and synthesis strategies of MXenes-based sodium-ion battery anode materials and the influence of various synthesis strategies on the structure and properties of MXenes-based materials are comprehensively summarized. Then, the first-principles research progress of MXenes-based sodium-ion battery anode materials is summarized, and the relationship between the storage mechanism and structure of sodium-ion batteries and the electrochemical performance is revealed. Finally, the key challenges and future research directions of the current design and synthesis strategies and first principles of these MXenes-based sodium-ion battery anode materials are introduced.

## 1. Introduction

In order to cope with the increasingly serious energy crisis and environmental pollution, people are gradually increasing the use of natural resources such as solar, wind and tidal energy; however, due to the complex distribution, discontinuity and other problems, people also need to find energy storage and conversion systems to convert these natural resources into clean energy that can be used by human beings continuously. Secondary batteries are an important class of energy storage devices. Since the commercialisation of lithium-ion batteries in 1991, they have played an important role in mobile electronics, electric vehicles and grid energy storage [1]. However, the high preparation cost, low energy density, and potential safety risks caused by organic electrolytes in lithium-ion batteries (LIBs) have triggered widespread concerns [2]; therefore, there is an urgent need to find alternative battery technologies for low-cost and high-safety applications to meet the stringent requirements of large-scale energy storage devices and a variety of consumer electronics products. Sodium-ion batteries have attracted great interest because they exhibit electrochemical properties similar to those of lithium-ion batteries, and sodium has the advantages of abundant reserves and low cost. However, compared with lithium, sodium has a larger ionic radius (0.102 nm), higher standard reduction potential (−2.71 V vs. standard hydrogen electrode (SHE)) and lower electronegativity (0.93), which leads to the fact that sodium-ion batteries always suffer from the slow adsorption and insertion of Na+ as well as a large volume expansion [3,4]. This results in sodium-ion batteries with a low reversible capacity and poor cycling stability, which limits the reversible capacity of sodium-ion batteries. The low reversible capacity and poor cycling stability of sodium-ion batteries limit their large-scale generation and use [5,6,7]. As a result, numerous researchers have focused on developing new sodium storage materials with tuneable chemical structures to improve sodium storage efficiency.

Since the successful exfoliation of graphene in 2004, two-dimensional (2D) materials have attracted great research interest due to their good properties, such as high electrochemical activity [8] and fast ion diffusion pathway [9]. In 2011, an emerging class of 2D materials, MXenes, was successfully prepared, and in the past 10 years, MXenes have been extensively studied, especially in the field of sodium-ion batteries. Extensive and in-depth research has been carried out on MXene materials, especially in the field of sodium-ion batteries. MXenes is a collective name for a class of 2D transition metal carbides or carbon-nitrides, whose general formula is denoted as M_n+1_X_n_T_x_, where M denotes the transition metal element (Ti, Nb, Sc etc.), X is the carbon or nitrogen element and T denotes the terminal surface groups (-O,-F and -OH), which are mainly formed by the use of Hydrofluoric acid or a mixture of hydrochloric acid and lithium fluoride prepared by selective etching of A-layers (Al, Si, and Ga etc.) from the corresponding M_n+1_X_n_T_x_ phase [10,11,12]. The highly conductive 2D lamellar structure [13] and tuneable surface chemistry [14] of MXene materials allow them to offer new possibilities as anode materials in solving the problems of slow Na^+^ diffusion rate and high diffusion barriers in sodium-ion batteries. However, the collapse and re-stacking of MXene lamellae hinder the full utilisation of MXenes’ active sites and limit the large-scale application of MXenes as anodes for sodium-ion batteries. Therefore, numerous researchers have promoted the further practical application of MXene materials in the field of energy storage devices by designing MXenes-based materials that can significantly increase the electronic conductivity and enhance their electrochemical performance while preventing the aggregation of individual nanosheets [15].

Since 2015, MXenes-based materials have developed rapidly in the field of energy storage, and a large number of research articles on sodium-ion batteries have been published. In this review, we first summarise the research work and development in the field of MXenes-based sodium-ion energy storage materials in recent years, focusing on the design and synthesis strategies of MXenes-based sodium-ion battery anode materials currently employed by, and in particular illustrating the effects of, the various design strategies on the structure and properties of MXenes-based materials, as shown in Table 1. On this basis, we also summarise the recent first-principles study of MXenes-based anode materials for sodium-ion batteries to reveal the storage mechanism and the relationship between the structure and electrochemical performance of sodium-ion batteries. Finally, the current challenges and future directions of the design and synthesis strategies and first-principles studies of MXene-based anode materials for sodium-ion batteries are summarised and outlined.

## 2. Design and Synthesis Strategy of Anode Materials for MXenes-Based Sodium-Ion Batteries

Although MXenes as an emerging anode material for sodium-ion batteries have attracted great research interest, their tendency to aggregate or stack in practical use greatly hinders electron transport, leading to their own low capacity and further limiting the large-scale application of MXenes. In order to solve these problems, researchers have proposed a number of design and synthesis strategies, which we summarise as elemental doping modification, binary material composite and ternary material composite.

### 2.1. Design and Synthesis of Elementally-Doped MXenes-Based Materials

**Figure 1:**
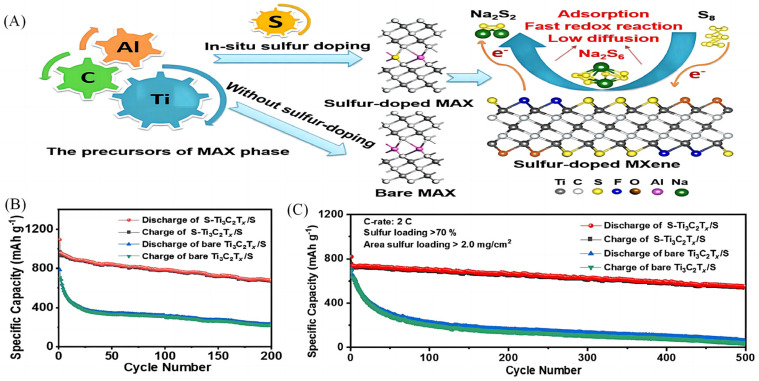

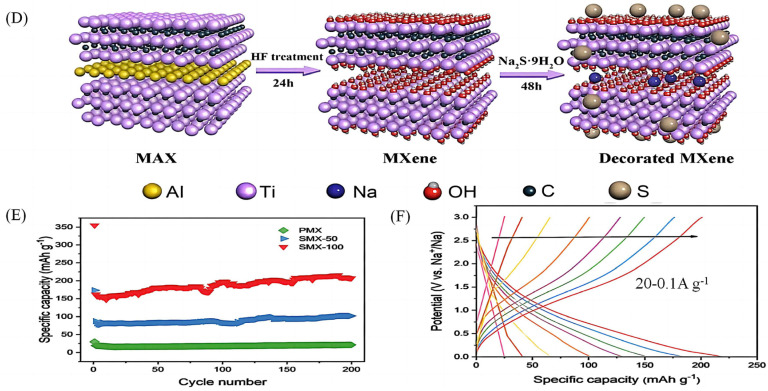
(**A**) Schematic diagram demonstrating the preparation of sulphur-doped MXene [17]. (**B**) Cycling performances of S-Ti_3_C_2_T_x_/S electrode and bare Ti_3_C_2_T_x_/S electrode at 0.5 C for 200 cycles [17]. (**C**) Cycling performances of S-T_i3_C_2_T_x_/S cathode and bare Ti_3_C_2_T_x_/S cathode at 2 C for 500 cycles [17]. (**D**) Schematic illustration of the preparation of sulphur-decorated Ti_3_C_2_ MXenes [18]. (**E**) Cycling performances of SMX-100 at 0.1 A g^−1^ [20]. (**F**) Charge–discharge curves at different current densities [20].

Elemental doping modification of MXene materials is one of the most common strategies employed by researchers to dope heteroatoms such as nitrogen (N) and sulphur (S) onto carbon-based materials [16] in order to enhance material specificity. Based on the conventional preparation of sulphur-doped multilayer Ti_3_C_2_T_x_ MXene, Bao et al. [17] prepared sulphur-doped Ti_3_C_2_T_x_ MXene (S-Ti_3_C_2_T_x_) with a 3D folded structure by employing a vacuum freeze-drying method (Figure 1A). The reduction and oxidation peaks of the negative electrodes of the S-Ti_3_C_2_T_x_ have a small potential difference between the reduced and oxidised peaks, and the enlarged interlayer spacing of the MXene leads to the electrodes with good dynamic properties. Its initial discharge capacity reached 970 mAh g^−1^, and the discharge capacity was maintained at 690.3 mAh g^−1^ after 200 cycles with a decay rate of 0.14% per cycle (Figure 1B). The capacity was maintained at 577.1 mAh g^−1^ after 500 cycles at 2C (Figure 1C). Sun et al. [18] produced sulphur-doped Ti_3_C_2_ MXenes by electrostatic self-assembly (Figure 1D), and the capacity of the electrodes of this material was 135 mAh g^−1^ after 1000 cycles at a current density of 2 A g^−1^, with an average capacity loss of only 0.033% per cycle. The average capacity loss per cycle was only 0.033%. The heat treatment temperature also has a certain effect on the elemental doping modification, and the S-doped MXene electrode has a larger interlayer spacing and more abundant active sites at high temperature [19]. Li et al. [20] used sulphur as a template to modulate the surface chemistry and microstructure of Ti_3_C_2_ calcined at 400 °C to obtain the mesoporous thin film of Ti_3_C_2_T_x_ (SMX-100), and the results of electrochemical studies showed that the electrode still maintained a stable specific capacity of 206.2 mAh g^−1^ after 200 cycles at 100 mA g^−1^ (Figure 1E), and the SMX-100 electrode consistently maintained a high capacitance during the charge/discharge cycling process (Figure 1F), which was mainly due to the effective penetration of the electrolyte and the increase in the layer spacing caused by the insertion of Na^+^. The results show that the incorporation of sulphur terminals can significantly accelerate the redox reactivity of Na–S cells and limit the outward diffusion of polysulfides, so that Na–S cells have higher rate capability.

**Figure 2:**
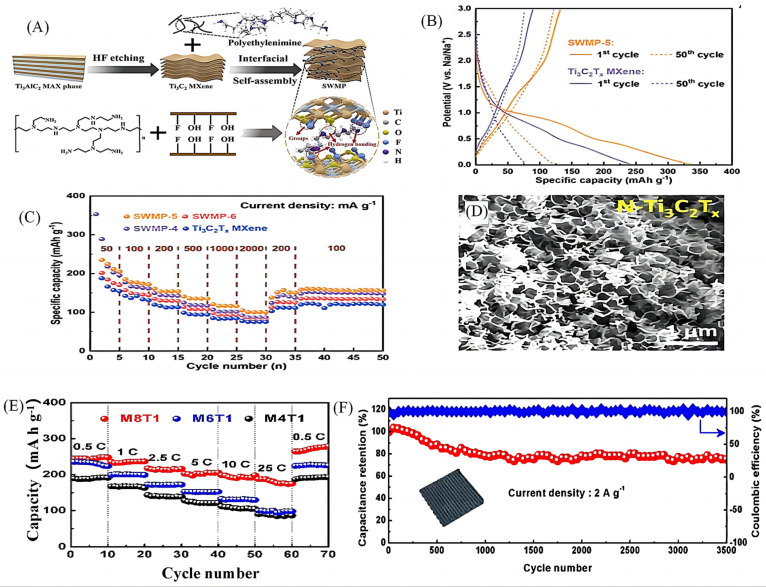
(**A**) Schematic illustration of the interfacial self-assembly MXene-based sandwich-like structure with polymer network [24]. (**B**) The initial and fiftieth cycle of galvanostatic charge/discharge curves of SWMP-5 and Ti_3_C_2_T_x_ MXene at 500 mA g^−1^ [24]. (**C**) Rate performance of SWMP-5 [24]. (**D**) SEM images of N-Ti_3_C_2_T_x_ (M8T1). (**E**) Rate performances of M8T1[25]. (**F**) Cycling performance of the 3D-printed SIC (SIC-4) at 2 A g^−1^. Inset: Real photo showing a 3D-printed woodpile N-Ti_3_C_2_T_x_ electrode [25].

Nitrogen doping has been shown to be one of the facile modification strategies to improve the electrochemical properties of MXenes [21,22,23]; however, the position of nitrogen doping has an effect on the electrical properties of MXenes due to the underlying mechanism. Jin et al. [24] synthesised Ti_3_C_2_ MXenes (SWMP-5) with sandwich structure by using the interfacial self-assembly method to ultrasonicate Ti_3_C_2_ in poly(vinyl imine) solution (Figure 2A). The dense N-rich polymer with a hydrogen bonding network connects the stable conductive framework to provide a fast channel for Na storage. The SWMP-5 negative electrode has an initial discharge capacity of 338.5 mAh g^−1^ (Figure 2B), and provides excellent multiplicative rates of 224.5, 185, 161, 141, 120, and 105 mAh g^−1^ at current densities ranging from 50–2000 mA g^−1^ capacity (Figure 2C). Fan et al. [25] used poly(melamine) microspheres as templates to treat Ti_3_C_2_ to obtain N-Ti_3_C_2_T_x_ (M8T1) with a highly uniform and well-defined porous framework structure (Figure 2D). The average capacity reaches 180.5 mAh g^−1^ (Figure 2E) at a current density of 25 C (1 C = 200 mA g^−1^), while the capacity retention after 3500 cycles at a current density of 2 A g^−1^ is 75% (Figure 2F), showing good cycling stability. The sodium-ion storage mechanism demonstrates that the interconnected nanosheets have a porous skeleton structure and high electronic conductivity, forming continuous conductive structures and creating multi-dimensional ion channels, which is conducive to rapid ion diffusion. The above studies show that surface modification of nitrogen atoms can greatly enhance the electrochemical reactivity and electronic conductivity of materials, and nitrogen doping strategies can induce well-defined porous structure, high surface area and macroporous volume. However, due to the influence of the underlying mechanism, the position of nitrogen doping has a certain influence on the electrical properties of MXenes, and the current research on its position is not comprehensive enough, so further research is still needed.

### 2.2. Design of MXenes-Based Binary Composite Synthesis

Compared with elemental doping, the design of- composite systems for MXenes can better solve the problems of slow ion transport due to the large radius of Na^+^ and volume expansion during electrochemical sodiation/desodiation [26,27,28]. Therefore, numerous researchers have focused on the design of MXenes-based binary composites to promote the embedding and de-embedding of Na^+^ during the electrochemical process of sodium-ion batteries and to improve the structural durability of the anode of sodium-ion batteries by constructing a binary heterojunction structure, which enhances the relevant performance of sodium-ion batteries. However, different preparation methods will not only affect the interaction between the second phase and MXenes, but also affect the morphology and properties of the composite. In this regard, we summarise the above adopted strategies as hydrothermal or solvent-thermal methods, electrostatic self-assembly methods, in situ synthesis methods and methods such as CVD.

#### 2.2.1. Hydrothermal Methods

**Figure 3:**
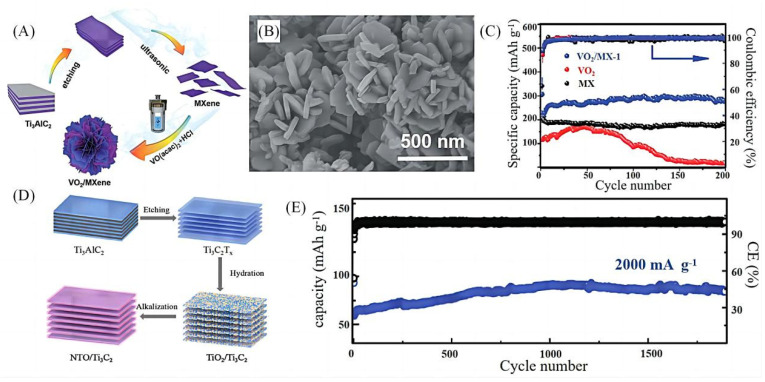

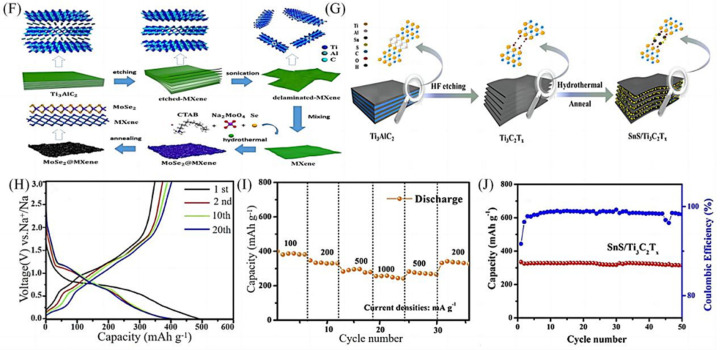
(**A**) Schematic illustration of the synthetic process for VO_2_/MXenes hybrids [29]. (**B**) SEM images of the VO_2_/MX-1[29]. (**C**) Cyclic performances of VO_2_/MX, VO_2_ and MX at a current of 0.1 A g^−1^ [29]. (**D**) Schematic illustration for synthesizing the NTO/Ti_3_C_2_ composite [30]. (**E**) Long-term cycling performance and CE data of the NTO/Ti_3_C_2_ anode at 2000 mA g^−1^ [30]. (**F**) illustration of the synthesis of single-layer MXenes and MoSe_2_/MXenes heterojunction [31]. (**G**) The SnS/Ti_3_C_2_T_x_ composites were synthesized by hydrothermal and annealing methods [32]. (**H**) The galvanostatic discharge/charge voltage profiles of the SnS/Ti_3_C_2_T_x_ composites at 100 mA g^−1^ [32]. (**I**) The rate capability of SnS/Ti_3_C_2_T_x_ electrode [32]. (**J**) The cycling performance of SnS/Ti_3_C_2_T_x_ composites with a current density of 500 mA g^−1^, and the corresponding coulombic efficiency [32].

The hydrothermal method is one of the most commonly used methods for the preparation of MXenes-based sodium-ion battery anode materials due to its advantages of easy preparation of nanomaterials with good crystallinity and different shapes and sizes. Wu et al. [29] prepared flower-like three-dimensional VO_2_/MXenes sodium-ion battery anode materials in different proportions (Figure 3B) by using the hydrothermal method (Figure 3A) with a 0.1 A g^−1^ current density after 200 cycles with a reversible capacity of 280.9 mAh g^−1^ (Figure 3C). Sun et al. [30] obtained NTO/Ti_3_C_2_ layered material with a porous structure on the basis of the MXenes’ stabilised structure by a two-step hydrothermal method (Figure 3D), and the composite material, after 1900 cycles at a current density of 2000 mA g^−1^, still provides a reversible capacity of 82 mAh g^−1^ (Figure 3E), which shows excellent stability.

Although the hydrothermal method is simple to operate and can produce a variety of novel and unique morphologies, there are still a series of shortcomings, such as high equipment requirements, long reaction time and high energy consumption. Based on this, more and more researchers are optimizing the hydrothermal method by combining two different preparation methods. Consequently, researchers have improved the hydrothermal method and combined it with other treatments to jointly prepare MXenes-based binary composites with better electrochemical properties, e.g., Xu et al. [31] used a simple hydrothermal method combined with a heat treatment process under H_2_ atmosphere to prepare a novel MoSe_2_/MXenes heterojunction ultrafast kinetic network electrode (Figure 3F). Afterwards, Zhang et al. [32] prepared SnS nanoparticle-modified Ti_3_C_2_T_x_ composites using a similar process (Figure 3G). The first-time charge/discharge capacities of SnS/Ti_3_C_2_T_x_ were 348.4 and 495.0 mAh g^−1^ (Figure 3I) with a coulombic efficiency of 70.4% (Figure 3H), and at a high current density of 1000 mA g^−1^. The discharge capacity was still maintained at 255.9 mAh g^−1^, and the reversible discharge capacity after 50 cycles at a current density of 500 mA g^−1^ was about 320 mAh g^−1^ with a Coulombic efficiency of 98% (Figure 3J). The use of CoNiO_2_, CoNi_2_O_4_, NiCo_2_O_4_, CoNi_2_S_4_ and other polytransition metal materials with high theoretical capacity and excellent electrical conductivity to enhance the electrochemical performance of MXene materials has also been an area of intense focus in recent years. Tao et al. [33] obtained CoNiO_2_/MXenes composites through the combination of the hydrothermal method and a heat treatment process, the first-time capacity of the material was 463 mAh g^−1^ and its reversible capacity reached 248.1 mAh g^−1^ in the charging/discharging experiment at 100 mA g^−1^. The good electrochemical performance of the CoNiO_2_/Ti_3_C_2_T_x_ composites is attributed to the increase in electrochemically active sites by the nano-CoNiO_2_ which shortens the diffusion paths of Na^+^ during the cycling process. Nevertheless, the long reaction time, complicated operation and high production cost of the hydrothermal method limits its use in large-scale production.

#### 2.2.2. Electrostatic Assembly Method

**Figure 4:**
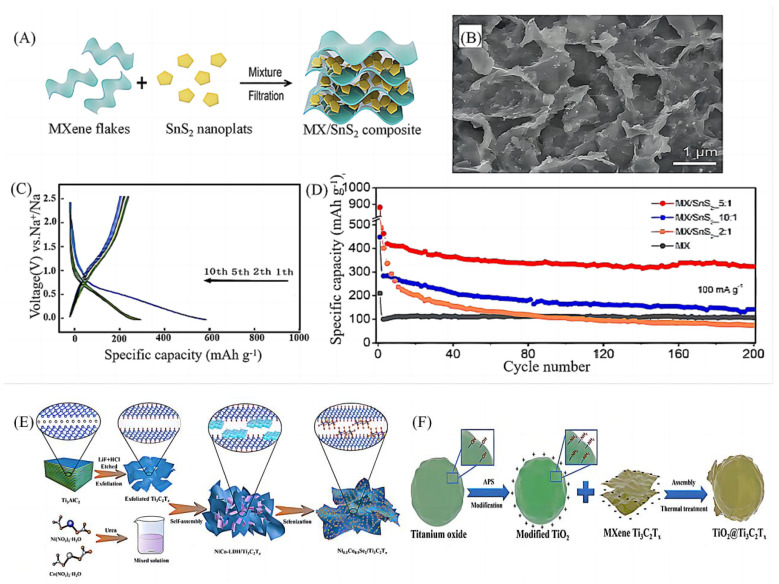
(**A**) The design concept and synthesis process of electrode materials for SIBs with high volumetric and gravimetric capacity [38]. (**B**) SEM images of MX/SnS_2_ [38]. (**C**) Galvanostatic charge-discharge profiles of MX/SnS_2_ 5:1 [38]. (**D**) Rate performance [38]. (**E**) The preparation process of the Ni_0.5_Co_0.5_Se_2_/Ti_3_C_2_T_x_ [39]. (**F**) Schematic illustration of synthetic process for TiO_2_@Ti_3_C_2_T_x_ material [40].

The self-assembly [34] method of electrochemical modification of the material surface has attracted much attention in the field of energy storage devices due to its simplicity, environmental friendliness, high efficiency, and low cost. Liu et al. [35] assembled VO_2_ nanotubes (VO_2_-NTs) and Ti_3_C_2_T_x_ MXenes into a multidimensional crosslinked structure of VO_2_-NTs/Ti_3_C_2_ anodes via electrostatic assembly. Xie et al. [36] prepared porous Ti_3_C_2_T_x_/CNTs composite thin films by electrostatic assembly combined with the chemical vapour deposition (CVD) method, which effectively prevented MXenes from being grafted in the structure. They also used the deposition (CVD) method to prepare porous Ti_3_C_2_T_x_/CNTs composite films, in which the grafted carbon nanotube CNTs in the structure effectively prevented the refolding of the MXene nanosheets and formed a porous structure, which facilitated the transport of the electrolyte and the access of ions to the electrodes. Zhao et al. [37] designed a novel molecular level PDDA-BP/Ti_3_C_2_ nanosheet heterostructure, which effectively discharged for more than 6 h to reach 3.0 V after 60 s of charging using a light-emitting diode (LED). Wu et al. [38], using the unique adsorption behaviours of oxygen-containing functional groups, produced MXenes that were self-assembled with SnS_2_ by vacuum-assisted filtration to obtain MXenes/SnS_2_ composites with different ratios (Figure 4A,B). The first charge/discharge capacities of the composites (MXenes/SnS_2_ = 5:1) at a current density of 100 mA g^−1^ were 460 mAh g^−1^ and 882 mAh g^−1^, respectively (Figure 4C), and the reversible capacity reached 322 mAh g^−1^ after 200 cycles (Figure 4D). In addition to the common lamellar MXenes-based composites, researchers have also constructed MXenes-based composites with a variety of microscopic morphologies, e.g., Dong et al. [39] used an in situ electrostatic attraction and selenide process to homogeneously anchor Ni_0.5_Co_0.5_Se_2_ nanoparticles to Ti_3_C_2_T_x_ conductive structures and prepared structurally stable Ni_0.5_Co_0.5_Se_2_/Ti_3_C_2_T_x_ composites (Figure 4E). Guo et al. [40] also prepared MXenes Ti_3_C_2_T_x_ nanosheet-encapsulated titanium oxide sphere structures (TiO_2_@Ti_3_C_2_T_x_) for the first time by using a self-assembly strategy (Figure 4F), and the MXene layer with high electronic conductivity protects the TiO_2_ spheres from electrochemical comminution, forming a stable solid-electrolyte interface. Although the electrostatic self-assembly technology is developing rapidly at present, there are some problems in its theoretical and practical applications, which urgently need to be further solved by researchers.

#### 2.2.3. In Situ Synthesis Method

**Figure 5:**
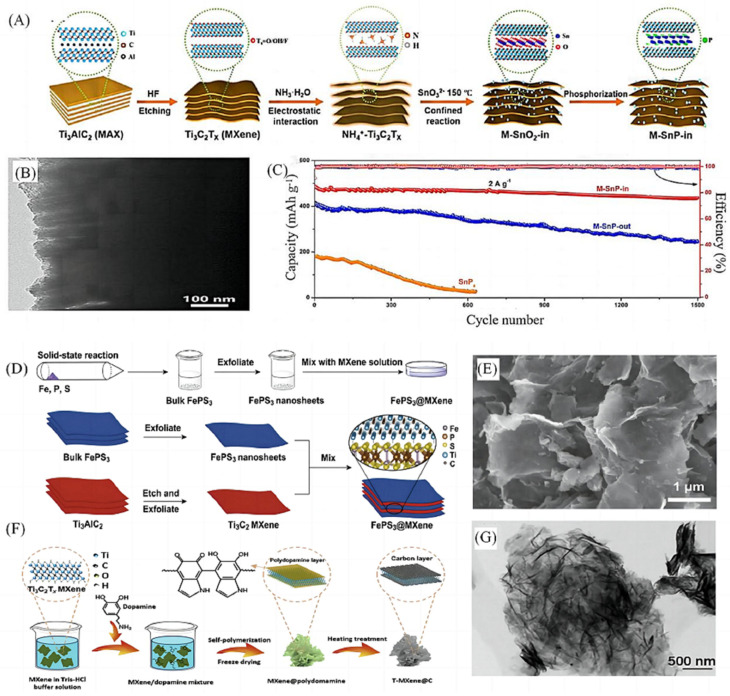
(**A**) Schematic illustration of the strategy for preparing M-SnP-in hybrids [41]. (**B**) Cycling performance tested at 2 A g^−1^ for 1500 cycles [41]. (**C**) Synthesis process diagram of FePS_3_@MXenes and Schematic illustration of MXenes assembled on FePS_3_ nanosheets surface and the micromolecular structure in enlarged view FePS_3_@MXenes [41]. (**D**) SEM images of FePS_3_@MXenes [42]. (**E**) Schematic diagram generalizing the preparation [42]. (**F**) Schematic diagram generalizing the preparation of T-MXene@C [44]. (**G**) Low-magnification TEM [44].

There are certain problems in the composite materials prepared by the adhesion method, such as the disadvantages of the introduced particle size, unstable thermodynamic properties and low interfacial bonding strength. Such problems can be ameliorated by in situ growth within the matrix through chemical reactions. In recent years, it has become one of the research hotspots to use in situ synthesis technology with the advantages of stable thermodynamic properties, non-polluting interfaces and high bonding strength to prepare MXenes-based binary composite anode materials, which are suitable for the construction of high-performance sodium-ion rechargeable batteries. Tang et al. [41] successfully prepared M-SnP-In composites through the growth of ultra-small SnO_2_ nanoparticles (NPs) in the intermediate layer of Ti_3_C_2_T_x_ composites (Figure 5A,B), and the M-SnP-In electrode maintained a high capacity of 436.6 mAh g^−1^ after 1500 cycles at a current density of 2A g^−1^ (Figure 5C), which is superior to most sodium-ion batteries reported so far. Ding et al. [42] successfully prepared FePS_3_@MXenes 2D/2D heterojunction composites by in situ mixing of ultrathin MXene nanosheets with FePS_3_ nanosheets (Figure 5D,E), and Ming et al. [43] prepared NaTiO_2_ nanotubes (NTO NTs) on Ti_3_C_2_F_x_ in situ as the enhanced sodium-ion battery. The NTO NTs were grown vertically on the Ti_3_C_2_F_x_ layer, which promoted Na^+^ diffusion and electron transfer and effectively inhibited the aggregation of the Ti_3_C_2_F_x_ layer. Zhang et al. [44] proposed a silver-ear-like carbon coated Ti_3_C_2_T_x_ (T-MXenes@C) anode material for sodium-ion batteries (Figure 5F,G). Self-polymerisation of dopamine on pristine Ti_3_C_2_T_x_ nanosheets effectively promoted the transformation of Ti_3_C_2_T_x_ into a silver-ear-like 3D structure, which was then carbonised in inert air to form a thin carbon coating that would protect the exposed surface of the Ti_3_C_2_T_x_ nanosheets from air oxidation and structural aggregation. The 3D silver-ear-like structure with active and stable surface (T-MXene@C) promotes charge transport, improved multiplicity performance and long cycling performance, among others. Despite the advantages of the in situ synthesis method, more in-depth studies on its reaction mechanism, practical applications and process optimisation are still needed to provide new possibilities for its industrial production.

Although the in situ synthesis method has many advantages, most of the in situ reaction systems are still in the stage of experimentation and development research, and the in situ reaction process is not easy to control, and the formation of intermediate phases cannot be controlled, which adversely affects the properties of materials. The content and ratio of reactants have a great influence on the formation of the reaction and the reaction speed, and it is difficult to control. It is still necessary to carry out more in-depth research on its reaction mechanism, practical application and process optimization to provide new possibilities for its industrial production.

#### 2.2.4. Other Synthesis Strategies

In addition to the synthetic methods involved above, there are some other synthetic strategies to prepare MXenes-based binary composite sodium-ion battery anode materials [45,46], such as chemical vapour deposition (CVD) and anodic oxidation. Shen et al. [47] successfully synthesised TiC/C core/shell nanowire arrays on Ti_6_Al_4_V substrates using a modified one-step chemical vapour deposition (CVD) method. The prepared TiC/C nanowire arrays have a core/shell structure and a porous structure, which increase the contact and active area, provide good buffering capacity against volume changes, and enable fast electron/ion transfer. Zhang et al. [48] successfully synthesised amorphous vanadium oxide/V_2_C MXenes (a-VO_x_/V_2_C) nano by anodic oxidation of multilayers of V_2_CT_x_ at a constant voltage in aqueous electrolyte hybrids. The reversible capacity was as high as 307 mAh g^−1^ at 50 mA g ^−1^, and the multiplicative capacity was as high as 96 mAh g^−1^ at 2000 mA g^−1^.

### 2.3. Design and Synthesis of MXene-Based Ternary Composites

**Figure 6:**
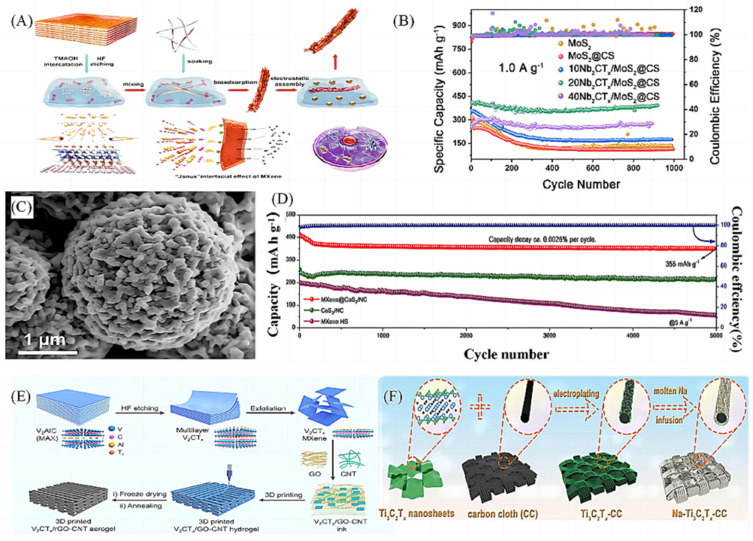
(**A**) Schematic illustration of transitional metal chalcogenide (TMC)on MXene coated fungal-derived carbonaceous nanoribbon (CNRib) heterostructure (TMC@MXene@CNRib) [49]. (**B**) Cycling performances at current density of 1 A g^−1^ [50]. (**C**) SEM images of MXene@CoS_2_/NC spheres in sequence [51]. (**D**) Long-term cycling stability of MXene@CoS_2_/NC [52]. (**E**) Schematic diagram of the preparation process of a 3D-printed V2CTx/rGO-CNT microgrid aerogel electrode [52]. (**F**) Schematic diagram of fabrication procedure for Ti_3_C_2_T_x_-CC skeletons and Na-Ti_3_C_2_T_x_-CC metal anodes [53].

Based on the synthetic basis of MXenes-based binary composites, researchers have designed and constructed MXenes-based ternary composites in anticipation of obtaining anode materials for sodium-ion batteries with better electrochemical properties. Cao et al. [49] then designed a unique microbial electrostatic assembly method based on the traditional electrostatic assembly method and prepared a 2D transition metal sulphide-MXene-carbon-based nanoribbon with heterogeneous structured negative electrodes (Figure 6A). This method promotes the migration and stable storage of high-capacity Na^+^ by constructing a heterojunction interface with multiple surface-active sites and highly conductive main chain structure, effectively alleviating the slow kinetics of sodium-ion batteries. However, the performance of MXenes-based composites prepared by a single synthesis process often fails to reach the expectation, and multiple preparation methods also need to be used together. Yuan et al. [50] used the template method and the electrostatic assembly method to prepare the Nb_2_CT_x_/MoS_2_@CS ternary composites, which had a specific capacity of 394 mAh g^−1^, with 74.1% capacity retention after 900 cycles at a current density of 1 A g^−1^, with good capacity stability (Figure 6B). Li et al. [51] combined the in situ growth method and template method to assemble Ti_3_C_2_T_x_ MXene nanosheets into thin-walled hollow spheres using PMMA spheres as sacrificial templates (Figure 6C), and then N-doped CoS_2_ nanoparticles embedded in them were grown to obtain MXene@CoS_2_/NC ternary composites. The electrochemical performance test shows that it has a high initial discharge capacity of 355 mAh g^−1^ and the capacity retention rate is around 100% after 5000 cycles (Figure 6D).

In addition to common preparation methods, some new synthesis processes have been gradually developed, and Wang et al. [52] innovatively proposed the preparation of a 3D printed V_2_CT_x_/rGO-CNT anode (Figure 6E). The hierarchical and porous 3D structure of this ternary composite not only maintains the relative integrity of the entire electrode structure, but also provides abundant active sites to enhance the Na ion/electron transport kinetics of sodium-ion batteries. Electrochemical performance tests showed that the anode material could maintain stable cycling for 3000 h at 2 mA cm^−2^ and 10 mAh cm^−2^ with an average Coulombic efficiency of 99.54%, as well as stable operation for 1000 h at high current densities of 5 mA cm^−2^ and 50 mAh cm^−2^. Fang et al. [53] have also used the novel synthesis processes-coating method and melt-infusion method to obtain Na-Ti_3_C_2_T_x_-CC ternary composites (Figure 6F). The composites have good anti-viscosity and machinability, which verifies the feasibility of producing flexible Na-metal electrodes and provides new possibilities to promote the practical application of sodium-ion batteries.

**Table 1:** Comparison of physical properties of MXene-based materials.

Serial Number	Material Composition	Preparation Method	First Charge (mAh g^−1^)	First Discharge (mAh g^−1^)	Cycling Performance	Rate Capability	References
1	S-doped Ti_3_C_2_T_x_	Vacuum freeze drying	821.7	970	577 mAh g^−1^ after 500 cycles		[17]
2	S-doped Ti_3_C_2_T_x_	Electrostatic self-assembly		325	135 mAh g^−1^ after 1000 cycles at 2.0 A g^−1^	136.6 mAh g^−1^ at 5 A g^−1^	[18]
3	S-doped mesoporous Ti_3_C_2_T_x_ film	Electrostatic self-assembly		354.6	345.6 mAh cm^−3^ after 5000 cycles at 1 A g^−1^	129.2 m Ah g^−1^ at 1 A g^−1^	[20]
4	N-doped Ti_3_C_2_T_x_	Electrostatic self-assembly	132.6	338.5	123.4 mAh g^−1^ after 5000 cycles at 1 A g^−1^	120 mAh g^−1^ at 1 A g^−1^	[24]
5	N-doped Ti_3_C_2_T_x_	Electrostatic self-assembly		1844.7	284.2 mAh g^−1^ after 1000 cycles at 5.0 C	180.5 mAh g^−1^at 25 C	[25]
6	VO_2_/MXenes	Hydrothermal method		229.2	280.9 mAh g^−1^ after 200 cycles at 0.1 A g^−1^	206 mAh g^−1^ at 1.6 A g^−1^	[29]
7	NaTi_8_O_13_/NaTiO_2_	Two-step hydrothermal method	125	162	82 mAh g^−1^ after 1900 cycles at 2.0 A g^−1^	143 mAh g^−1^ at 0.1 A g^−1^	[30]
8	MoSe_2_/MXene	Hydrothermal method combined with thermal annealing process	578	826	384 mAh g^−1^ after 400 cycles at 2.0A g^−1^	490 mAh g^−1^ at 1.0A g^−1^	[31]
9	SnS/Ti_3_C_2_T_x_	Hydrothermal method combined with thermal annealing process	348.4	495.0	320 mAg^−1^ after 50 cycles at 500 mAg^−1^	255.9 mAh g^−1^ at 1000 mA g^−1^	[32]
10	CoNiO_2_/MXene	Hydrothermal method combined with thermal annealing process		463	223 mAh g^−1^ after 140 cycles at 0.1 A g^−1^	188 mAh g^−1^ at 0.3 A g^−1^	[33]
11	VO_2_-NTs/Ti_3_C_2_	Electrostatic self-assembly	1164	2132	516 mAh g^−1^ after 2000 cycles at 5.0 A g^−1^	703 mAh g^−1^ at 10.0 A g^−1^	[35]
12	Ti_3_C_2_T_x_/CNT	Electrostatic self-assembly	179	501	120 mAh g^−1^ after 500 cycles at 0.1 A g^−1^		[36]
13	PDDA-BP/Ti_3_C_2_	Electrostatic self-assembly	1780	2588	1112 mAh g^−1^ after 500 cycles at 0.1 A g^−1^	560 mAh g^−1^ at 1 A g^−1^	[37]
14	TiO_2_@Ti_3_C_2_T_x_	Electrostatic self-assembly	233.9	497.1	116 mAh g^−1^ after 5000 cycles at 0.96 A g^−1^	177 mAh g^−1^ at 0.12 A g^−1^	[40]
15	M-SnP-In	In situ synthesis		438.2	436.6 mAhg^−1^ after 1500 cycles at 2 Ag^−1^	438.2 mAhg^−1^ at 15 A g^−1^	[41]
16	T-MXene@C	In situ synthesis	580.6		499.4 mAh g^−1^ after 200 cycles at 0.2 C	478 mAh g^−1^ at 0.2 C (1 C = 320 mA g^−1^)	[44]
17	a-VO_x_/V_2_C	In situ synthesis		161	54 mAh g^−1^ after 1800 cycles at 2000 mA g^−1^	96 mAh g^−1^ at 2 Ah g^−1^	[48]
18	Cu_1.75_Se—MXene—CNRib	Microbial electrostatic assembly	744.2	1353.1	305.6 mAh g^−1^ after 400 cycles at 1.0 A g^−1^	435.3, 356.2, 315.7, 274.3, 232.6, and 161.3 mAh g^−1^ at 0.1 to 5.0 A g^−1^	[49]
19	Nb_2_CT_x_/MoS_2_/CS	Electrostatic self-assembly method + template method		1270	526 mAh g^−1^ after 100 cycles at 0.1 A g^−1^ 394 mAh g^−1^ after 900 cycles at 1 A g^−1^	196 mAh g^−1^ at 20 A g^−1^	[50]
20	MXene@CoS_2_/NC	In situ growth method + template method	660	885	620 mAhg^−1^ after 200 cycles at 0.2 A g^−1^	708, 614, 551, 438, and 394 mAh g^−1^ at 0.2, 0.5, 1, 2, and 5 A g^−1^	[51]

## 3. First-Principles Study of Anode Materials for MXenes-Based Sodium-Ion Batteries

The first principles based on density functional theory can simulate the kinetics of electrochemical reactions from the atomic level, which helps to understand and analyse the relevant energy storage mechanism of battery materials, and also provides a theoretical basis for the design of new battery materials, which is one of the important research tools in the field of energy storage devices. In the research process of MXenes-based anode materials for sodium-ion batteries, many researchers have conducted many studies on the electronic structure of MXene materials, the structure and physical and chemical properties of element-doped MXene materials and MXenes-based composites before and after modification, which have made great contributions to the understanding of the role of MXene materials in sodium-ion batteries.

All first-principles calculations were performed by the Vienna Ab initio Simulation Package (VASP). We chose projected-augmented-wave method (PAW) to describe the electron-ion interaction. The exchange–correlation interaction functional is the generalized gradient approximation (GGA) in the Perdew–Burke–Ernzerhof (PBE) functional.

### 3.1. MXenes Theoretical Computational Study of the Electronic Structure of the Material

**Figure 7:**
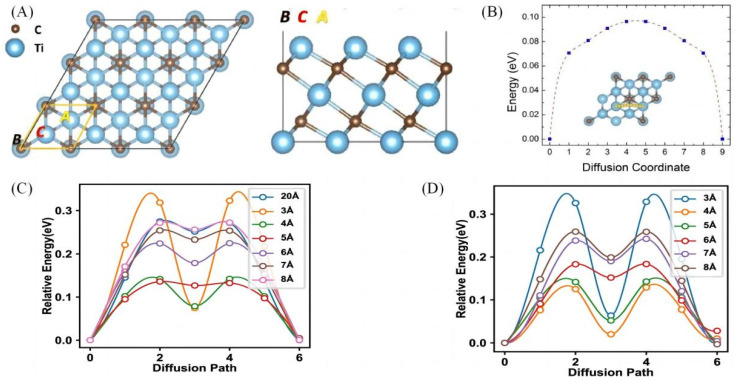
(**A**) Schematic diagram showing the crystal structure of a Ti_3_C_2_ monolayer with top and side view. The large blue balls represent Ti atoms and the small brown balls represent C atoms. The highlighted unit cell indicates the high-symmetry A, B, and C adatom [54]. (**B**) Schematic representation of the top view of the energetically optimized migration pathways and the corresponding diffusion barrier profiles of Na [54]. (**C**) Distribution of the energy barrier profiles of Na diffusion under Different interlayer spacings in C–Ti stacking of Ti_3_C_2_O_2_ [55]. (**D**) Distribution of the energy barrier profiles of Na diffusion under Different interlayer spacings in C-Ti stacking of C-Tistacking of Ti_2_CO_2_ [55].

Er et al. [54] conducted a first-principles study of the electronic structure of sodium ions on Ti_3_C_2_ in order to understand the differences in their adsorption on Ti_3_C_2_. Through the calculation of Na adsorption energy (Figure 7A), the adsorption energy was found to be only 4.40 eV, and combined with the results of density of states simulation of Ti_3_C_2_ before and after Na adsorption, the diffusion barrier of Na^+^ was 0.096 eV (Figure 7B), which indicated that Ti_3_C_2_ is a promising anode material for sodium-ion batteries. The basic mechanism for the MXene column effect to enhance the electrode performance of sodium-ion batteries is still unclear; based on this, Dai et al. [55] investigated the effects of different MXene interlayer stacking methods on the adsorption, diffusion and mechanical properties of Na atoms by employing the first nature principle with two typical MXenes as the research objects. It was found that for both Ti_3_C_2_O_2_ and Ti_2_CO_2_ configurations, the diffusion energy barriers of Na were greatly reduced when the C-Ti stacking mode was adopted (Figure 7C,D). This provides a useful theoretical basis for understanding the MXene column effect and contributes to the development of anode materials for sodium-ion batteries. This was followed by a first-principles study of the Na storage capacity of Ti_3_C_2_T_x_ by Bai et al. [56], which, after comprehensive calculations for all configurations of Eads2, extended the calculations to their theoretical capacities and obtained the optimal capacity-to-structure ratios in the stacked structure. The calculated CMNa value can be increased from 218.32 mAh g^−1^ to 413.13 mAh g^−1^, which is in good agreement with the previously reported value of 413.0 mAh g^−1^. The analysis of the optimal capacity-to-structure ratio shows that the increase in layer spacing favours the increase in MXene capacity, which provides a theoretical basis for achieving high-capacity sodium storage behaviour.

### 3.2. Theoretical Computational Study of Elementally Doped MXenes Materials

**Figure 8:**
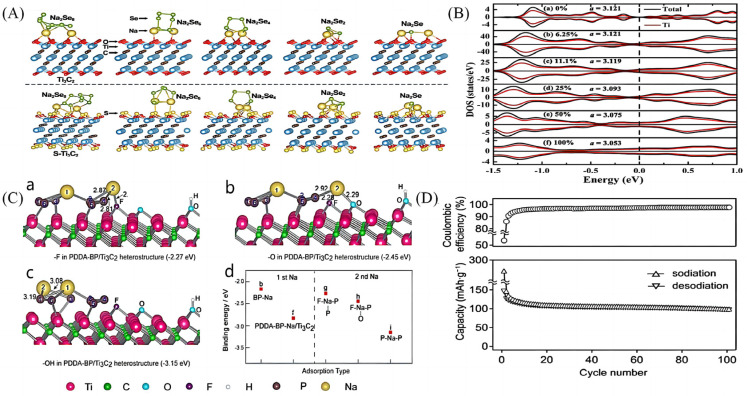
(**A**) Adsorption configurations of Na_2_Se_n_ (n = 8, 6, 4, 2, and 1, respectively) on the surface) and pristine Ti_3_C_2_ [57]. (**B**) Total and partial density of states (DOS) of N-doped Ti_2_C monolayer with doping concentrations of (a) 0%, (b) 6.25%, (c) 11.1%, (d) 25%, (e) 50% and (f) 100%, i.e., Ti_2_N monolayer. a is the lattice constant for a per unit cell at different concentrations of nitrogen [59]. (**C**) (**a**–**c**) the most stable adsorption configurations for another Na adsorption on top of functional groups in the heterostructures, such as -F, -O and -OH. (**d**) The calculated binding energies for Na adsorption on the surface of BP nanoparticle or PDDA-BP/Ti_3_C_2_ heterostructures with different surface functional groups [37]. (**D**) Cycle performance for Ti_3_C_2_T_x_ at 20 mA g^−1^ [61].

Using elemental doping as one of the common modification means, researchers have carried out a lot of studies on the first nature principle of elementally doped MXene materials. Bao et al. [17] experimentally demonstrated that the S-modified Ti_3_C_2_ material has a strong adsorption of Na_2_S_n_ in the Na–S cell. Based on this, Lu et al. [57] used a first-principles simulation to calculate the chemical properties and bond strengths between S-modified Ti_3_C_2_ and Na_2_Se_n_, which showed that S-doped Ti_3_C_2_ has strong anchoring and trapping effects on Na_2_Se_n_ (Figure 8A). S-doped MXenes not only improve the chemical bonding energy and van der Waals force on Na_2_Se_n_, but also, more importantly, the joint action of S-doped MXene materials and the nano-scaled Na_2_Se_n_ together can maximize the surface or near-surface charge storage of MXene materials, which is conducive to improving the charging and discharging capacity of sodium-ion batteries. Wang et al. [58], on the other hand, systematically investigated the performance of sulphur-functionalized MXenes as anode materials for sodium-ion batteries. The structural, kinetic and electronic properties of S-functionalized Ti_2_C single molecule membranes (Ti_2_CS_2_) were comprehensively investigated by first-principles calculations. The results show that the Ti_2_CS_2_ monolayers have not only good kinetic stability, but also metallic properties, thus ensuring excellent structural stability and electronic conductivity. Meanwhile the low diffusion barrier of Na^+^ and multilayer stable adsorption ensure the excellent capacity of Ti_2_CS_2_ monolayers in sodium-ion (935.57 mAh g^−1^) batteries, which is a theoretical basis for the design of high-capacity sodium-ion batteries. Liao et al. [59] investigated the effect of N doping on the electrical conductivity of Ti_2_C and Ti_3_C_2_ MXene materials before and after N doping using first-principles calculations. The results show that N doping can significantly improve the electrical conductivity of Ti_2_C and Ti_3_C_2_ (Figure 8B), while both Ti_2_C and Ti_3_C_2_ exhibit metallicity at different N doping concentrations, which offers the possibility of their further application in sodium-ion batteries. In order to achieve the performance of fast charging and long cycling in sodium-ion batteries, Xia et al. [60] explored the effect of N doping on the electronic structure of Ti_3_C_2_ using first-principles calculations. The results show that N atom doping induces charge redistribution and the formation of active sites in the MXene material, and at the same time improves the electronic conductivity of Ti_3_C_2_O_1.83_N_0.17_,which suggests the feasibility of realising high-performance sodium-ion electrodes based on the MXene material at room temperature.

In addition to the common studies on N and S-element-doped MXene materials, researchers have also investigated the first principles of other element-doped MXene materials. Zhao et al. [37] investigated the relaxation process when Na was added to the surface functional groups (-F, -O, and -OH), and by comparing the binding energy as well as the charge densities (Figure 8C), the researchers found that the intercalated Na^+^ first binds to the BP nanoparticles and then undergoes mixed adsorption on -F, -O and -P, which consequently reduces the binding energy and thus facilitates the diffusion of Na^+^ and charge transfer. Kajiyama et al. [61] have revealed that Ti_3_C_2_T_x_ exhibits a reversible Na^+^ embedding/de-embedding behaviour into/from the interlayer space in the non-Na^+^ electrolyte without a structural reason for the structural changes. The interlayer distance was maintained throughout the sodiation/desorption process due to the column-supporting effect of the captured Na^+^ and the swelling effect of the permeated solvent molecules between the Ti_3_C_2_T_x_ sheets, achieving reversible embedding/de-embedding of Na^+^, and thus no substantial structural changes occurred during the electrochemical reaction. For these reasons, Ti_3_C_2_T_x_ shows good capacity retention as well as excellent multiplicity in 100 cycles (Figure 8D), making it a promising anode material for sodium-ion batteries.

### 3.3. Theoretical Computational Study of MXenes Matrix Composites

Composite modification of MXene materials is also a good means of enhancing the properties, so many researchers have also focused on first-principles-related studies on MXenes-based composites. Zhao et al. [37] studied the interaction between Na and BP nanoparticles by first-principles calculations, and found that the interaction between Na and BP nanoparticles was significantly altered by comparing the Na-BP system and the PDDA-BP/Ti_3_C_2_ system before and after the relaxation process in terms of conformation, binding energy and charge density, it was found that Ti_3_C_2_ nanosheets significantly changed the sodic state of BP nanoparticles, which made it easier for them to obtain electrons from Na, facilitated the sodic process, and improved the sodium storage performance of the MXenes-based anode materials. Cao et al. [49] have conducted first-principles computational studies on the diffusion at the interface of MSe-MXenes-CNRib heterostructure behaviour, which was investigated by first-principles calculations. By studying the density of states of the ternary heterostructure, the adsorption energy, the difference in charge distribution of the Na adsorption heterostructure, and the energy distribution state of diffusion, it was not only confirmed that the synergistic effect would promote the adsorption and diffusion of Na ions to a certain extent, but it was also found that the heterostructure was capable of high-capacity storage of Na ions under different multiplicity conditions. Liu et al. [35] created a rational construction of the MXenes-based composite system based on the density functional theory, and the VO_2_ nanotubes (VO_2_-NTs) and Ti_3_C_2_T_x_ MXenes were composed into the VO_2_-NTs/Ti_3_C_2_ negative electrode material with a multidimensional cross-linking structure. Relevant theoretical calculations show that the rich multidimensional channels of the composite reduce the diffusion impedance of Na^+^, while the enhanced intrinsic conductivity and highly rigid structure solve the problems of poor cycling stability and multiplicity performance of VO_2_ due to the large volume change, which promotes the further practical application of sodium-ion batteries.

## 4. Conclusions

This review summarises the research efforts and developments in recent years on the design and synthesis strategies as well as first-principles studies of MXenes-based anode materials for sodium-ion batteries, detailing their numerous achievements in the field of sodium-ion batteries, and providing new possibilities for promoting the further practicalisation of sodium-ion batteries. In spite of the great progress made, there are still several key issues that have not been resolved in the research on the design and synthesis strategies and first principles of MXene-based anode materials for sodium-ion batteries, as shown below:

(1) Currently, the main method to obtain MXenes is still acid etching, which is characterised by low yield and high risk. Therefore, there is an urgent need for environmentally friendly, safe, efficient and high-quality methods, such as CVD or PVD, to synthesize MXenes with a controllable number of layers, adjustable surface groups, increased layer spacing and excellent quality. In addition, there is a need to optimize the existing synthesis methods and conduct in-depth research on the generation mechanism, which can be used to promote the practical large-scale industrial production of MXenes and MXenes-based materials.

(2) As mentioned earlier, the surface chemistry of MXenes may significantly affect the properties of MXenes-based materials. Different etchants can produce groups with different surface chemistries, but the precise surface chemistry of MXenes and the interactions between the surface groups and the materials are still not well understood. In addition, it was shown that bare/-O capped MXenes have better physicochemical properties. Therefore, more studies are needed to modulate the surface chemistry and explore its application in electrochemical storage.

(3) Another major problem with MXenes is their stability. The interlayer structure of an MXene is unstable under room temperature conditions, which can easily lead to the problem of heavy stacking, which seriously hinders the long-term development of MXenes-based materials. To alleviate this situation, efforts can be focused on rational structural design and precise morphological control of electrodes, such as 3D porous structure, aerogel and coating technology.

(4) Theoretical studies must take into account the inhomogeneous and incomplete mixed coverage of surface groups and MXene-stacked multilayers in order to accurately predict their properties and thus guide experiments. It is thus necessary to combine theoretical calculations with advanced computations, including machine learning, classical molecular dynamics and high-throughput calculations, to fully understand the surface chemistry and interfaces of MXenes-based materials. In addition, relevant theoretical models as well as computational software and hardware should be further developed to narrow the gap between theoretical predictions and experimental results.

In summary, MXenes are one of the electrode materials with great potential for development in the field of energy storage devices, but they still face great challenges in moving from the laboratory research stage to the industrial application stage. This requires researchers to conduct sufficient practical studies to improve the knowledge and understanding of this class of materials in order to explore and exploit its material advantages and to promote the commercial application of MXenes.

## Data Availability

All relevant data are provided in the article.
